# Based on machine learning, CDC20 has been identified as a biomarker for postoperative recurrence and progression in stage I & II lung adenocarcinoma patients

**DOI:** 10.3389/fonc.2024.1351393

**Published:** 2024-07-24

**Authors:** Rui Miao, Zhi Xu, Tao Han, Yafeng Liu, Jiawei Zhou, Jianqiang Guo, Yingru Xing, Ying Bai, Zhonglei He, Jing Wu, Wenxin Wang, Dong Hu

**Affiliations:** ^1^ School of Medicine, Anhui University of Science and Technology, Huainan, China; ^2^ Anhui Province Engineering Laboratory of Occupational Health and Safety, Anhui University of Science and Technology, Huainan, China; ^3^ Institute of Precision Medicine (AUST-IPM), Anhui University of Science and Technology, Huainan, China; ^4^ Department of Clinical Laboratory, Anhui Zhongke Gengjiu Hospital, Hefei, China; ^5^ School of Public Health, Anhui University of Science and Technology, Huainan, China; ^6^ Key Laboratory of Industrial Dust Prevention and Control & Occupational Safety and Health of the Ministry of Education, Anhui University of Science and Technology, Huainan, China

**Keywords:** machine learning, lung adenocarcinoma, recurrence, invasion, CDC20

## Abstract

**Objective:**

By utilizing machine learning, we can identify genes that are associated with recurrence, invasion, and tumor stemness, thus uncovering new therapeutic targets.

**Methods:**

To begin, we obtained a gene set related to recurrence and invasion from the GEO database, a comprehensive gene expression database. We then employed the Weighted Gene Co-expression Network Analysis (WGCNA) to identify core gene modules and perform functional enrichment analysis on them. Next, we utilized the random forest and random survival forest algorithms to calculate the genes within the key modules, resulting in the identification of three crucial genes. Subsequently, one of these key genes was selected for prognosis analysis and potential drug screening using the Kaplan-Meier tool. Finally, in order to examine the role of CDC20 in lung adenocarcinoma (LUAD), we conducted a variety of *in vitro* and *in vivo* experiments, including wound healing assay, colony formation assays, Transwell migration assays, flow cytometric cell cycle analysis, western blotting, and a mouse tumor model experiment.

**Results:**

First, we collected a total of 279 samples from two datasets, GSE166722 and GSE31210, to identify 91 differentially expressed genes associated with recurrence, invasion, and stemness in lung adenocarcinoma. Functional enrichment analysis revealed that these key gene clusters were primarily involved in microtubule binding, spindle, chromosomal region, organelle fission, and nuclear division. Next, using machine learning, we identified and validated three hub genes (CDC45, CDC20, TPX2), with CDC20 showing the highest correlation with tumor stemness and limited previous research. Furthermore, we found a close association between CDC20 and clinical pathological features, poor overall survival (OS), progression-free interval (PFI), progression-free survival (PFS), and adverse prognosis in lung adenocarcinoma patients. Lastly, our functional research demonstrated that knocking down CDC20 could inhibit cancer cell migration, invasion, proliferation, cell cycle progression, and tumor growth possibly through the MAPK signaling pathway.

**Conclusion:**

CDC20 has emerged as a novel biomarker for monitoring treatment response, recurrence, and disease progression in patients with lung adenocarcinoma. Due to its significance, further research studying CDC20 as a potential therapeutic target is warranted. Investigating the role of CDC20 could lead to valuable insights for developing new treatments and improving patient outcomes.

## 1 Introduction

Lung cancer ranks as one of the most prevalent and deadly diseases globally, accounting for 11.6% of new cases and 18.4% of mortality rates each year (1).Non-small cell lung cancer (NSCLC) accounts for approximately 80% of all cases based on its pathological classification (2). Despite efforts to improve clinical outcomes through various treatment strategies such as surgery, radiotherapy, chemotherapy, and immunotherapy, the prognosis remains unsatisfactory, with early metastasis being a significant factor contributing to high mortality rates (3). In recent years, targeted therapy based on molecular alterations has been successfully applied in various cancers, including breast cancer (4), ovarian cancer (5), and NSCLC (6). This encourages the search for additional biomarkers that could serve as potential targets for therapy.

Studies have shown that the recurrence and invasion of lung cancer are closely associated with various molecular mechanisms. For example, mutated tumor suppressor genes like TP53 (7) and RB1 (8) can lead to dysregulation of cell apoptosis and proliferation. Abnormal DNA methylation and histone modifications (9) can cause changes in transcription factors and tumor suppressor gene expressions, promoting the recurrence and invasion of lung cancer. Some studies have suggested a potential association between the recurrence and invasion of lung cancer and the existence of cancer stem cell subpopulations within the tumor. These stem cells possess self-renewal and multi-directional differentiation abilities, enabling them to reestablish the tumor and promote recurrence and invasion (10). Therefore, targeted therapy directed at lung cancer stem cells may help control the recurrence and invasion of lung cancer. Additionally, modulation of the tumor microenvironment may also contribute to the suppression of recurrence and invasion. Factors such as immune cells, fibroblasts, and angiogenesis within the tumor microenvironment play significant roles in lung cancer recurrence and invasion. For instance, the polarization status and infiltration level of tumor-associated macrophages are closely associated with the prognosis of lung cancer (11).

Targeted therapies directed at specific biomarkers or signaling pathways have shown promise in treating recurrence and invasion of lung cancer. For example, lung cancer patients with EGFR mutations can benefit from treatment with EGFR inhibitors, which can inhibit tumor recurrence and invasion (12). Other targeted therapies such as anti-angiogenic drugs and immune checkpoint inhibitors have also demonstrated potential therapeutic effects (13, 14). However, there are still challenges in effectively targeting tumor metastasis, highlighting the need for a deeper understanding of the molecular mechanisms underlying tumor metastasis. This will help identify more effective treatment approaches and improve the success rate and overall survival of lung cancer patients (15, 16).

This study aims to comprehensively identify key genes associated with recurrence, invasion, and tumor stemness using Weighted Gene Co-expression Network Analysis (WGCNA) and explore their corresponding biological functions. Subsequently, various algorithms were employed to further investigate hub genes. Finally, we focused on the gene CDC20 to explore its impact on malignant phenotypes of tumor cells both *in vitro* and *in vivo* and elucidate its underlying mechanism.

## 2 Materials and methods

### 2.1 Data download and preprocessing

First, mRNA expression profiles and clinical information of GSE166722 (n=53), GSE31210 (n=226), GSE30219 (n=182), GSE50081 (n=125), and GSE42127 (n=284) datasets were obtained from the Gene Expression Omnibus (GEO) database (GEO, https://www.ncbi.nlm.nih.gov/GEO/). Additionally, mRNA expression data and clinical information of the TCGA-LUAD cohort were acquired from the UCSC Xena browser (https://xenabrowser.net/). In the GSE166722 dataset, 53 cases of early-stage lung adenocarcinoma were classified into indolent tumors (ais and mia) and invasive tumors (iac) based on pathological grading. The GSE31210 dataset provided complete information on recurrence status and disease-free survival time for stage I&II patients. Therefore, the primary analysis was mainly based on GSE166722 and GSE31210, while the GSE30219, GSE50081, GSE42127, and TCGA-LUAD datasets were used as validation sets. Additionally, mutation information of the LUAD cohort from TCGA database was downloaded.

### 2.2 Identification of core gene modules associated with invasion and recurrence and functional enrichment analysis based on WGCNA

We utilized the WGCNA R package (v1.68) to identify the gene co-expression networks associated with invasion and recurrence in the GSE166722 and GSE31210 datasets. Hub genes within specific modules were defined based on gene significance (GS), where the Pearson correlation of each gene with invasion and recurrence > 0.2, and module membership (MM) > 0.8. Subsequently, the clusterprofiler R package (v3.14.3) was employed to perform Gene Ontology (GO) and Kyoto Encyclopedia of Genes and Genomes (KEGG) analyses on the hub genes within the co-expression modules.

### 2.3 The random forest and random survival forest algorithms were used to screen for important candidate genes associated with invasion and recurrence

The random survival forest and random forest are machine learning algorithms commonly used for dimensionality reduction analysis. The identified gene set associated with invasion and recurrence was included in the random survival forest analysis, using the “randomForest” and “randomSurvivalForest” packages in R, selecting the top 30 genes. Key genes were obtained by analyzing the overlapping genes using a Venn diagram. Subsequently, the effectiveness of these key genes was assessed through survival analysis and the construction of ROC curves.

### 2.4 Malignant pathological features of key genes

The prognostic role of key genes in several independent LUAD cohorts was analyzed using Kaplan-Meier curve analysis. The correlation between key genes and clinical pathological features, including pathological stage, TNM stage, age, and gender, was assessed using the chi-square test. The prognostic independence of key genes in predicting Overall Survival (OS) and Progression-Free Interval (PFI) was determined using the univariate and multivariate Cox regression analysis.

#### 2.4.1 Molecular characteristics related to CDC20

In the previous study (17), we obtained a set of stemness genes and quantified them using the single-sample gene set enrichment analysis (ssGSEA) method. We analyzed their correlation with CDC20 using the Pearson algorithm. We also applied an algorithm to analyze the carcinogenic pathways in patients and assess their correlation with CDC20 using the Pearson algorithm. Subsequently, a differential analysis of CDC20 (using the median value as a threshold) was performed on six independent LUAD cohorts. Differentially expressed genes with an absolute log2 fold change greater than 1 were selected as criteria, and the intersecting genes were identified. These genes were visualized using the STRING database (https://cn.string-db.org/). Finally, the mutation status of these genes was visualized using a waterfall plot.

#### 2.4.2 Potential drug screening for CDC20-related targets

Using oncoPredict package (https://cran.r-project.org/web/packages/oncoPredict/index.html), with known cell line filter data to predict or cancer drug reactions in the body and biomarkers, IC50 values of eight drugs were obtained.

Differential gene analysis was performed by dividing patients into high and low groups based on the median expression of CDC20 in the GSE30219, GSE31210, GSE42127, GSE50081, GSE166722, and TCGA datasets. The resulting differentially expressed genes were intersected. These differentially expressed genes were then imported into the Connectivity Map (CMAP) database (https://clue.io/about) to screen for potential therapeutic drugs associated with CDC20.

### 2.5 Cell culture and siRNA transfection

Cell lines derived from Anhui University of Science and Technology School of Medicine were used in this study. All cell lines were authenticated using short tandem repeat (STR) analysis. Human lung epithelial cells BEAS-2B,the lung cancer cell lines A549, H1975, and H1299, were cultured in DMEM medium containing 10% fetal bovine serum (FBS) and 1% penicillin-streptomycin (Biological Industries, Israel) at 37°C in a 5% CO_2_ incubator. A549, H1975, and H1299 cells were seeded in plates and transfected with siRNA using Lipofectamine 2000 (Invitrogen, USA) when the cells reached 70% confluency. The siRNA reagents were purchased from GenePharma.The siRNA sequences were as follows: si_CDC20#1 (S: 5’-CGCCUGAAAUCCGAAAUGATT-3’, AS: 5’-UCAUUUCGGAUUUCAGGCGTT-3’); si_CDC20#2 (S: 5’-CACAGAACCAGCUAGUUAUTT-3’, AS: 5’-AUAACUAGCUGGUUCUGUGTT-3’); si_CDC20#3 (S: 5’-CCACCAAGAAGGAACAUCATT-3’, AS: 5’-UGAUGUUCCUUCUUGGUGGTT-3’); at the same time, we used a scramble sequence that did not specifically target any gene transfected cells as the NC group, si_CDC20#NC(S: 5’-UUCUCCGAACGUGUCACGUTT-3’, AS: 5’-ACGUGACACGUUCGGAGAATT-3’), we used this as a siRNA control targeting CDC20.

### 2.6 Western blot

SiRNA transfected H1975 and H1299 cells were collected 24h after transfection, and RIPA lysis buffer was added to extract cell proteins on ice. The protein samples were subjected to 10% sodium dodecyl sulfate-polyacrylamide gel electrophoresis (SDS-PAGE) and then transferred onto a PVDF membrane. The membrane was blocked with 5% skim milk for 1 hour and incubated overnight at 4°C with specific primary antibodies (diluted 1:1000). The specific primary antibodies used were as follows: CDC20 (PTG, 10252-AP), ERK (Servicebio, GB11004-100), JNK (Servicebio, GB114321-100), P38 (Servicebio, GB113380-100), and GAPDH (ABclonal, AC001). After washing the PVDF membrane three times with TBS-T solution for 10 minutes each, chemiluminescent detection was performed, and the protein band intensity was analyzed using Image J software.

### 2.7 Real-time quantitative PCR analysis

Total RNA was extracted from cultured cells using Trizol reagent (Invitrogen) according to the manufacturer’s instructions. RNA extracted from each group was used for reverse transcription through the Revert Aid First Strand cDNA Synthesis Kit (Thermo Scientific). The obtained cDNAs were used for real-time quantitative PCR (RT-qPCR) through 2X Universal SYBR Green Fast QPCR Mix system. The results were analyzed in a QuantStudio 3 Real-Time PCR System (quantstudio design & analysis software, Thermo Scientific).

### 2.8 Wound healing assay

The H1975 and H1299 cell suspensions were added to a 6-well plate and placed in a cell incubator. Once the cells formed a confluent monolayer, the cells were transfected with siRNA. After transfection for 6h,a sterile pipette tip (200μL) was used to create scratch wounds. Subsequently, the wells were washed three times with PBS. Images of the scratch wounds were captured at 0h and 24h using an inverted microscope.Use serum-free medium after scratch.

### 2.9 Colony formation experiment

SiRNA transfected H1975, H1299 cells were collected 24h after transfection and cultured in 6-well plates at a density of 2000 cells per well for 7-10 days. The medium was changed every three days. After colonies formed, the colonies were fixed, washed, and stained to observe clone formation. The colonies were counted using Image J software, and statistical analysis was performed.

### 2.10 Transwell migration assay

The upper compartment was filled with 100μL of 1%BSA DMEM cell suspension containing 1 × 10^4^ H1975 or H1299 cells collected 24 hours after transfection of siRNA.Additionally, 600μL of complete culture medium was added to the lower chamber. The chamber was incubated at 37°C with 5% CO_2_ for 24 hours. Subsequently, the chamber was removed and the cells were fixed in 4% paraformaldehyde for 20 minutes. Excess cells in the upper chamber were gently wiped with a cotton swab, washed twice with PBS, and stained with crystal violet for 20 minutes. Finally, the cells were observed under an inverted microscope, and random images were captured.

### 2.11 Cell flow cytometry for cell cycle analysis

The cell cycle was determined using propidium iodide (PI) DNA staining. H1975 and H1299 cells were seeded in cell culture dishes and incubated at 37°C with 5% CO_2_ for 24 hours. The cells were then treated with siRNA and incubated again at 37°C with 5% CO_2_ for an additional 24 hours. After isolating the cells, they were centrifuged, washed with PBS, fixed with 75% cold ethanol, and stored overnight at 4°C. The cells were washed with PBS, treated with RNase and PI, and incubated at 37°C for 60 minutes. Flow cytometry analysis was performed using a flow cytometer, with an excitation wavelength of 488 nm and an emission wavelength of 610 nm. The collected data from 10,000 cells were analyzed using Modfit 5.0 software.

### 2.12 Construction of sh-CDC20 LA-4 cell stability

One day prior to transfection, HEK-293 (Pricella) cells were seeded to reach a density of 60% to 80%. Six hours before transfection, the cells were switched to a non-complete culture medium. 2μg of sh-CDC20 lentiviral plasmid were diluted in 400 μl of Opti-MEM (ThermoFisher Scientific, #31985070), and simultaneously, 8 μl of Lipofectamine 2000 were diluted in another 400 μl of Opti-MEM. Both solutions were gently mixed and allowed to stand for 5 minutes. Then, the Lipofectamine 2000 mixture was slowly added to the plasmid solution and allowed to stand for an additional 10 minutes. Subsequently, the combined solution was added to the cell culture plate and gently mixed. The cells were cultured for 6 hours at 37°C with 5% CO2, followed by replacement with fresh DMEM medium. After 24 hours, the expression of ZsGreen protein in HEK-293 cells was observed using a fluorescence microscope to assess the efficiency of plasmid transfection.

### 2.13 Heterotransplantation tumor growth

We selected sh-CDC20 LA-4 stable cells to verify the effect of CDC20 on tumor formation in mice. Healthy male C57BL/6 mice, aged 4-6 weeks, were randomly divided into control and shCDC20 groups. Subcutaneously injected (100μl) into the armpit of the forelimb of mice, and the cell density was 1 × 10^8^/ml.The mice’s health condition and normal food intake were observed. Tumor size was measured using calipers, and on the 28th day, the mice were euthanized using cervical dislocation, and the tumors were completely dissected. Sh group and NC group, 5 animals in each group.Tumor size and volume were calculated using the formula: V = (L × W^2^)/2, where L and W represent the length and width of the subcutaneous tumor, respectively. The animal experiments were approved by the Medical Ethics Committee of Anhui University of Science and Technology.

### 2.14 Statistical analysis

All independent experiments were repeated three times, and the results were analyzed using GraphPad Prism software. Independent sample t-test and Wilcoxon signed-rank test were used between the two groups; one-way ANOVA (One-way ANOVA) and multiple comparisons were used between the three groups to compare the control and experimental groups. Statistical significance is denoted as follows: ns, not significant; * p < 0.05; ** p ≤ 0.01; *** p ≤ 0.001.

## 3 Results

### 3.1 Identification of key gene groups related to invasion and recurrence using WGCNA

**Figure 1:**
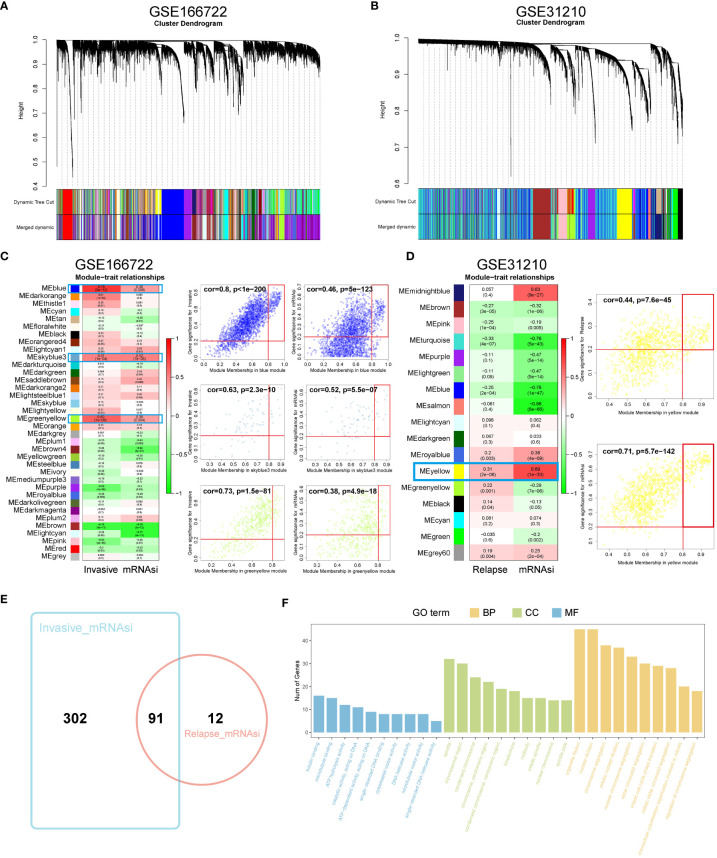
Identification of WGCNA module genes. **(A, B)** In the GSE166722 and GSE31210 datasets, a clustering dendrogram was formed using weighted correlation coefficients to group genes with similar expression patterns into co-expression modules, with each module represented by a different color. **(C, D)** In the GSE166722 and GSE31210 datasets, correlation heatmaps illustrating the relationship between module characteristic genes (MEs) and invasion/recurrence, as well as scatter plots showing the correlation between module membership (MM) and gene significance (GS), were generated. **(E)** Venn diagram of invasion and recurrence. **(F)** KEGG analysis and GO enrichment analysis of the module genes.

WGCNA was used to identify gene modules highly correlated with invasion and recurrence in the GSE166722 and GSE31210 datasets (**Figures 1A, B**). The blue (cor = 0.8, P < 1e-200), sky blue (cor = 0.63, P = 2.3e-10), and yellow-green (cor = 0.73, P = 1.5e-81) modules in the GSE166722 dataset were found to be associated with invasion. Similarly, the blue (cor = 0.46, P = 5e-123), sky blue (cor = 0.52, P = 5.5e-07), and yellow-green (cor = 0.38, P = 4.9e-18) modules showed correlation with the mRNA stemness index (mRNAsi) score (**Figure 1C**). In the GSE31210 dataset, the yellow module (cor = 0.44, P = 7.6e-45) was selected as key genes for further analysis based on MM > 0.8 and GS > 0.2 (**Figure 1D**). Intersection of genes from these modules resulted in the identification of 91 hub genes (**Figure 1E**). Functional enrichment analysis using Gene Ontology (GO) revealed their association with microtubule binding, spindle, chromosomal region, organelle fission, and nuclear division (**Figure 1F**).

### 3.2 Machine learning is used to screen key genes for recurrence and invasion

**Figure 2:**
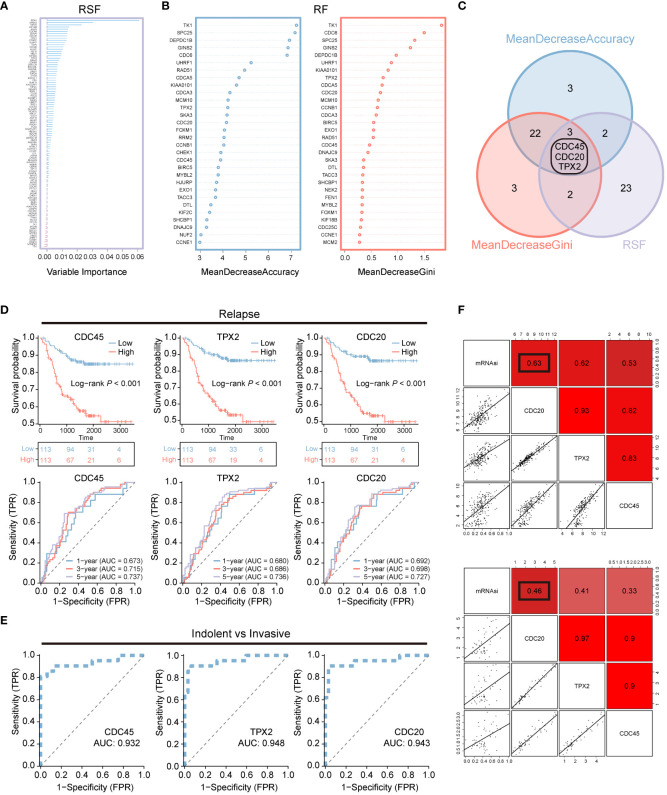
Machine learning is used for screening recurrent and invasive key genes. **(A–C)** Random Survival Forest and Random Forest are two algorithms along with their overlapping Venn diagram. **(D)** OS Kaplan-Meier survival curve analysis was performed for the high and low expression groups of three genes in the LUAD patients cohort GSE31210. Additionally, ROC curves depicting the time-dependent changes showed the area under the curve (AUC) values for patient OS at 1 year (blue), 3 years (red), and 5 years (purple). **(E)** The ROC curve illustrates the diagnostic efficacy of CDC45, TPX2, and CDC20 in differentiating between invasive and non-invasive LUAD patients within the GSE166722 cohort. **(F)** The correlation between CDC20, TPX2, and CDC45 with stemness mRNAsi.

To further reduce the dimensionality of genes, we employed random survival forests and intersected their results, yielding three hub genes: CDC45, CDC20, and TPX2 (**Figures 2A–C**). Concurrently, we analyzed the impact of CDC45, CDC20, and TPX2 expression on patient OS and 1-, 3-, and 5-year predictive abilities in the GSE31210 cohort. We found that high expression of these three genes led to poorer prognosis but better survival prediction capabilities (**Figure 2D**). Moreover, in the GSE166722 cohort, all three genes demonstrated strong predictive abilities for high invasion (**Figure 2E**).

Additionally, we assessed the correlation between CDC45, CDC20, and TPX2 with tumor stemness using Pearson correlation analysis (**Figure 2F**). We found that the correlation between CDC20 and stemness was 0.63 and 0.46 in both datasets, respectively, which was higher than that of CDC45 (0.62,0.41) and TPX2 (0.53,0.33).

### 3.3 The prognostic significance and malignant clinical characteristics of CDC20 in patients with lung adenocarcinoma

**Figure 3:**
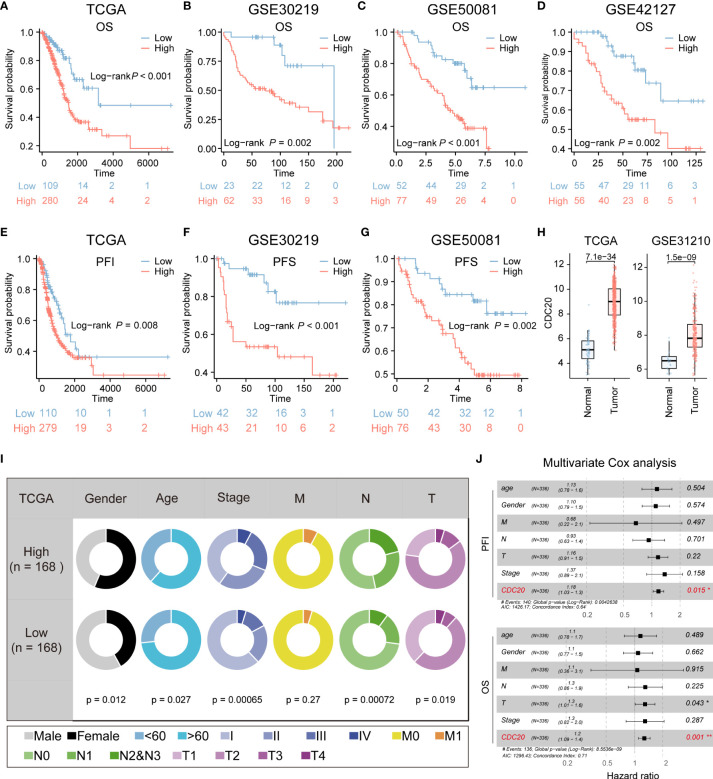
CDC20 is significantly associated with aggressive clinical phenotype in LUAD. **(A–D)** The OS curves of patients with high and low expression of CDC20 were analyzed in the TCGA, GSE30219, GSE50081, and GSE42127 cohorts. **(E, F)** Kaplan-Meier curves of PFI in patients with high and low expression of CDC20 in the TCGA cohort and PFS in patients with high and low expression of CDC20 in the GSE30219 cohort. **(G–H)** PFS curves of patients with high and low expression of CDC20 were analyzed in the GSE50081 cohort. Furthermore, box plots were generated to depict the differential expression of CDC20 between normal tissue and tumors in the TCGA and GSE31210 cohorts. **(I)** The clinical characteristics of patients with high and low expression of CDC20 in the TCGA cohort. **(J)** A multivariable Cox regression analysis was performed to further screen for the key cancer gene CDC20 in LUAD using the TCGA database.

Due to the relatively high correlation between CDC20 and tumor stemness, and limited research on the association between CDC20 and postoperative recurrence and invasion in lung adenocarcinoma, subsequent studies primarily focused on CDC20. The prognostic value of CDC20 mRNA expression was evaluated in the Kaplan-Meier Plotter database, and overall survival (OS) curves were plotted for LUAD patients from the TCGA, GSE30219, GSE50081, and GSE42127 cohorts. As shown in **Figures 3A–D**, higher mRNA expression of CDC20 was significantly associated with a poorer prognosis in LUAD patients. Additionally, progression-free interval (PFI) curves from TCGA data and progression-free survival (PFS) curves from GSE30219 and GSE50081 data were plotted (**Figures 3E–G**). The results revealed that patients with high CDC20 expression had shorter progression-free intervals and progression-free survival compared to those with low CDC20 expression. Furthermore, a comparison of CDC20 expression between normal and tumor tissues in TCGA and GSE31210 cohorts showed significantly higher expression in tumor tissues (**Figure 3H**). These findings suggest a significant association between CDC20 mRNA expression and the prognosis of LUAD patients, highlighting its potential as a useful biomarker for predicting patient survival.

Additionally, the combined analysis of clinical and pathological features demonstrated that patients with high CDC20 expression tended to exhibit more advanced pathological stages (**Figure 3I**). CDC20 may serve as a potential therapeutic target for LUAD treatment. Multivariable Cox regression analysis further conducted on the TCGA-LUAD cohort indicated that CDC20 could be an independent prognostic factor in LUAD (**Figure 3J**). Overall, these results strongly suggest a high correlation between CDC20 and tumor malignancy.

### 3.4 The molecular characteristics of tumors with high expression of CDC20 and identification of related gene clusters

**Figure 4:**
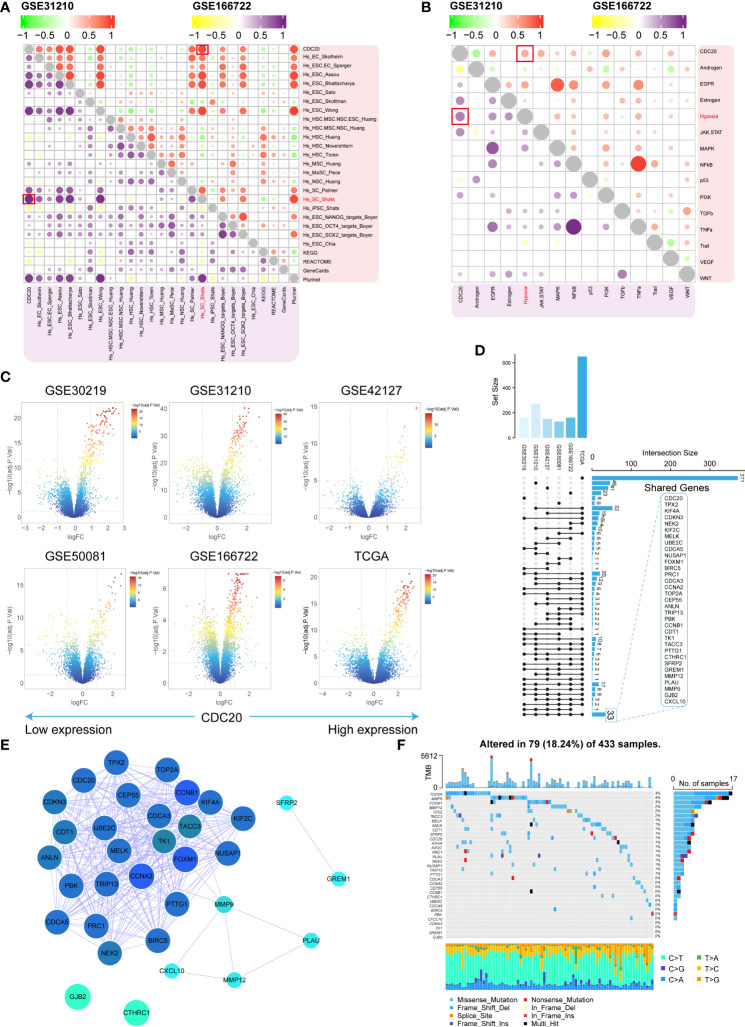
Molecular characteristics and identification of related gene clusters. **(A, B)** The stemness gene sets and associated oncogenic pathways positively correlated with CDC20 in the GSE31210 and GSE166722 datasets are highlighted in red boxes. **(C)** Volcano plot displaying the distribution of differentially expressed genes in the GSE30219, GSE31210, GSE42127, GSE50081, GSE166722, and TCGA datasets. **(D, E)** Shared gene sets and PPI network analysis across the six datasets. **(F)** Waterfall plot illustrating the highest mutation rates in the top 33 genes based on stemness.

CDC20’s association with stemness was assessed in the GSE31210 and GSE166722 datasets. Positive correlations were observed between CDC20 and 26 stemness gene sets (**Figure 4A**). Subsequently, oncogenic pathways in these datasets were screened, revealing a positive correlation between CDC20 and the Hypoxia pathway in both datasets (**Figure 4B**). Differential analysis was further performed on six datasets (GSE30219, GSE31210, GSE42127, GSE50081, GSE166722, TCGA) to explore 33 key genes, including CDC20 (**Figures 4C, D**). Subsequently, a protein-protein interaction (PPI) network analysis was conducted on these genes to assess their protein-level interactions, as shown in **Figure 4E**. Additionally, the mutation landscape of these key genes was visualized (**Figure 4F**). Collectively, these results partially elucidate the molecular characteristics of tumors with high CDC20 expression.

### 3.5 The relationship between CDC20 and drug sensitivity was analyzed, and the potential drugs were screened by related gene groups

**Figure 5:**
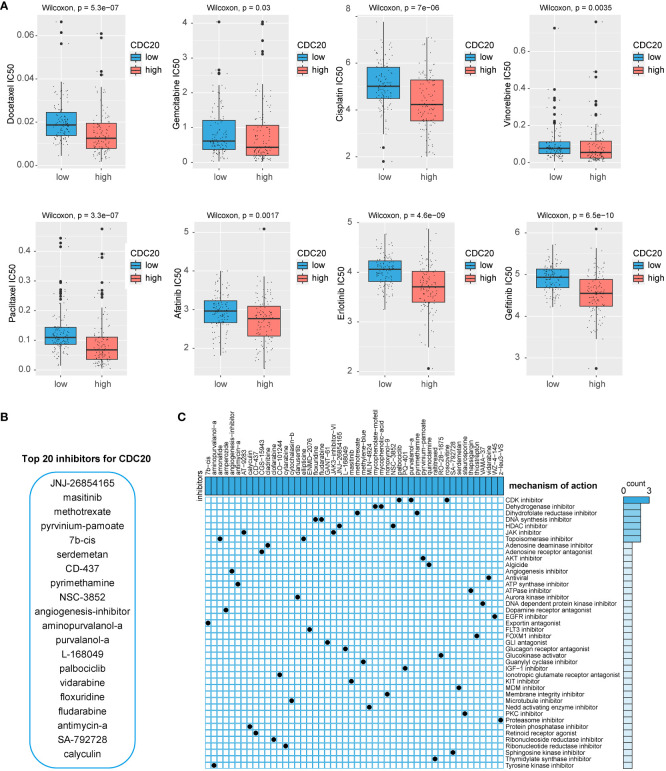
Potential drug screening. **(A)** IC50 values of 8 chemotherapy drugs. **(B)** CAMP screening for potential inhibitors of CDC20. **(C)** Potential drug screening for CDC20-related gene clusters.

To identify potential drugs highly sensitive to CDC20 expression, this study utilized predictive algorithms to estimate the chemotherapeutic sensitivity of chemotherapy drugs and compared them between high and low CDC20 expression groups. As shown in **Figure 5A**, the estimated IC50 values of Docetaxel, Gemcitabine, Cisplatin, Vinorelbine, Paclitaxel, Afatinib, Erlotinib, and Gefitinib were significantly lower in the high CDC20 expression group across each LUAD dataset, suggesting that individuals with high CDC20 expression might exhibit higher sensitivity to these drugs. Subsequently, the study employed CAMP visualization to screen and prioritize the top 20 drugs targeting CDC20 scores, including JNJ-26854165, masitinib, methotrexate, pyrvinium-pamoate, 7b-cis, serdemetan, CD-437, pyrimethamine, NSC-3852, angiogenesis inhibitor, aminopurvalanol-a, purvalanol-a, L-168049, palbociclib, vidarabine, floxuridine, fludarabine, antimycin-a, SA-792728, and calyculin (**Figure 5B**). Additional drug screening was performed for the gene clusters obtained from (**Figures 4E**), revealing potential drug categories identified by CAMP that could serve as a foundation for targeted therapies (**Figure 5C**).

### 3.6 CDC20 knockdown inhibits cell migration, tumor invasion, and proliferation

**Figure 6:**
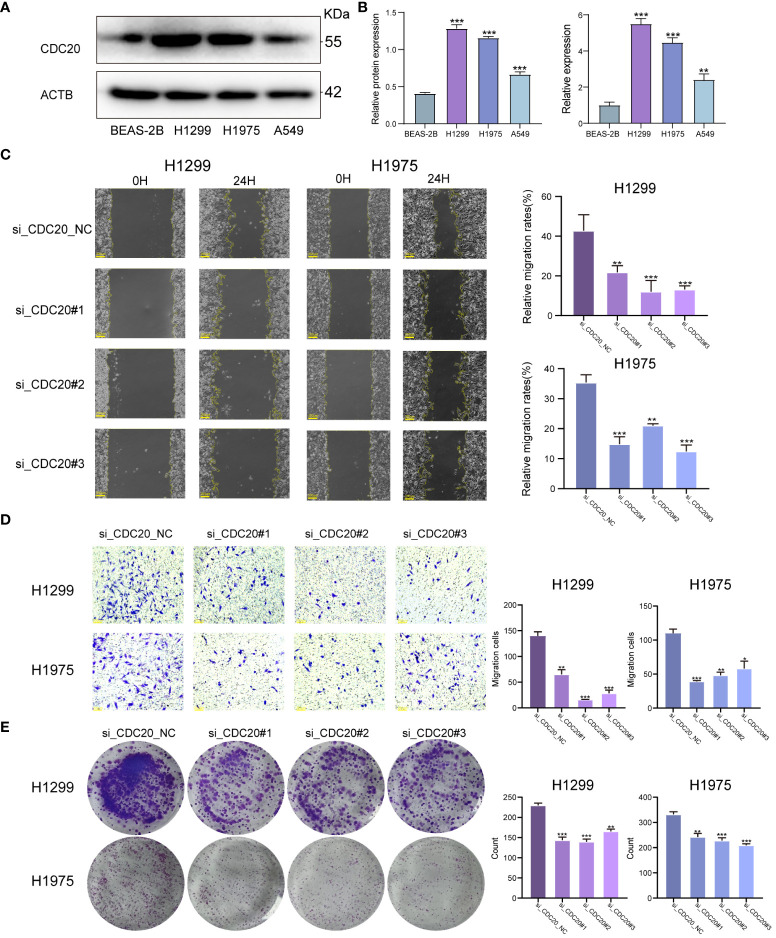
CDC20 promotes migration, invasion and proliferation of lung adenocarcinoma cells *in vitro.***(A, B)** CDC20 protein expression levels and mRNA levels were verified in three lung cancer cell lines and normal epithelial cells. **(C)** Images of wound healing in the NC and CDC20 knockdown groups of H1299 and H1975 lung cancer cells are presented. **(D)** Images from the Transwell assay are shown for the NC and CDC20 knockdown groups of H1299 and H1975 lung cancer cells. **(E)** Colony formation images are provided for the NC and CDC20 knockdown groups of H1299 and H1975 lung cancer cells. (***p < 0.001; **p < 0.01; *p < 0.05).

To clarify the promotional effect of CDC20 on lung adenocarcinoma, our study initially verified the expression levels of CDC20 protein and mRNA in human epithelial cell line BEAS-2B and three lung cancer cell lines, H1299, H1975, and A549. We found that the protein and mRNA expression levels of CDC20 were significantly higher in all three lung cancer cells compared to the normal epithelial cells, with H1299 and H1975 cells showing relatively higher expression (**Figures 6A, B**). As the level of CDC20 protein expressed in A549 cells was relatively low, it was considered futile to knock down CDC20 in these cells. Consequently, to more effectively elucidate the impact of CDC20 reduction on migration and invasion behavior, we selected the H1299 and H1975 cell lines, which exhibited higher levels of CDC20 expression, for knockdown experiments.

We did the Wound healing assay and Transwell migration assay.In the Wound healing assay, we analyzed the migration rate at 24 hours. The average migration rate of H1299 cells in the NC group at 24 hours was 42.7%, with a standard deviation (SD) of 8. After knocking down CDC20, the migration rates decreased significantly to 21.8%, 13.8%, and 13.1%, with SD values of 3, 4, and 1, respectively. Similarly, after the same knockdown treatment on H1975 cells, the average 24-hour migration rate in the NC group was 35.4%, with an SD of 4. In the knockdown group, the 24-hour migration rates were 14.9%, 21%, and 12.4%, with SD values of 4, 1, and 3, showing a significant decrease in migration. Therefore, we can conclude that knocking down CDC20 in both cell types inhibits and significantly reduces cell migration ability. Additionally, in the Transwell migration assay, the average number of cells passing through the chamber for H1299 cells in the NC group and knockdown group were 139, 65, 16, and 28, respectively. For H1975 cells, the numbers were 110, 39, 48, and 58.The results from the Wound healing assay and Transwell migration assay demonstrated a significant inhibition of migratory and invasive abilities in lung cancer cells upon CDC20 knockdown (**Figures 6C, D**). In the colony formation assay, we obtained that the mean colonies of single cell clone formation in the NC group and knockdown group of H1299 cells were 229,143,139,165, respectively, and that in H1975 cells were 331,242,227,208, respectively (**Figure 6E**). Additionally, the colony formation assay showed decreased proliferative ability in H1299 and H1975 cells following CDC20 knockdown.In conclusion, these findings provide strong evidence supporting the crucial role of CDC20 in promoting the malignant characteristics of lung adenocarcinoma.

### 3.7 Knockdown of CDC20 affects the cell cycle and is associated with the MAPK pathway

**Figure 7:**
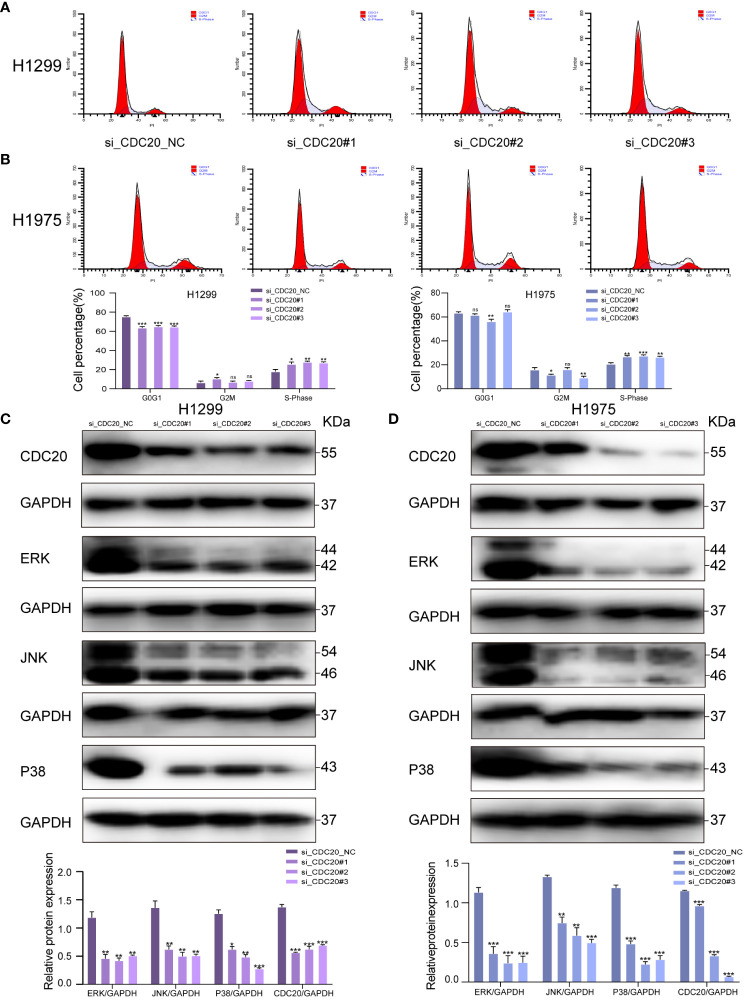
CDC20 affects the cell cycle and may be associated with the MAPK signaling pathway. **(A, B)** Flow cytometry of H1299 and H1975 cells in NC group and CDC20 knockdown group. **(C, D)** To verify the expression of MAPK signaling pathway by Western blotting assay in H1299 and H1975 cells transfected with siRNA. (***p < 0.001; **p < 0.01; *p < 0.05).

To elucidate the potential mechanistic role of CDC20 in tumor malignancy, we transfected H1299 and H1975 cells with siRNA. Flow cytometry analysis revealed that the knockdown group of H1299 and H1975 cells exhibited arrest at the S phase, indicating an impact on the cell cycle (**Figures 7A, B**). To further investigate the potential mechanisms of CDC20 in LUAD, we discovered a significant enrichment of the MAPK signaling pathway upon CDC20 knockdown based on other literature references (18).

In addition, previous research has reported that the activation of the MAPK pathway promotes the malignant progression of LUAD (19). Thus, we hypothesize that CDC20 may play a role in promoting LUAD through the MAPK signaling pathway. To investigate this hypothesis, we conducted Western blotting analysis to determine the impact of CDC20 knockdown on the expression of MAPK nodes in both H1299 and H1975 cells. The results revealed a significant downregulation of ERK1/2 (p-ERK1/2), p38 (p-p38), and JNK (p-JNK1/2) protein levels following CDC20 knockdown (**Figures 7C, D**). These findings suggest that CDC20 has an influence on the cell cycle and may potentially contribute to the progression of LUAD through the ERK/MAPK signaling pathway.

### 3.8 CDC20 knockdown inhibited tumor formation in mice

**Figure 8:**
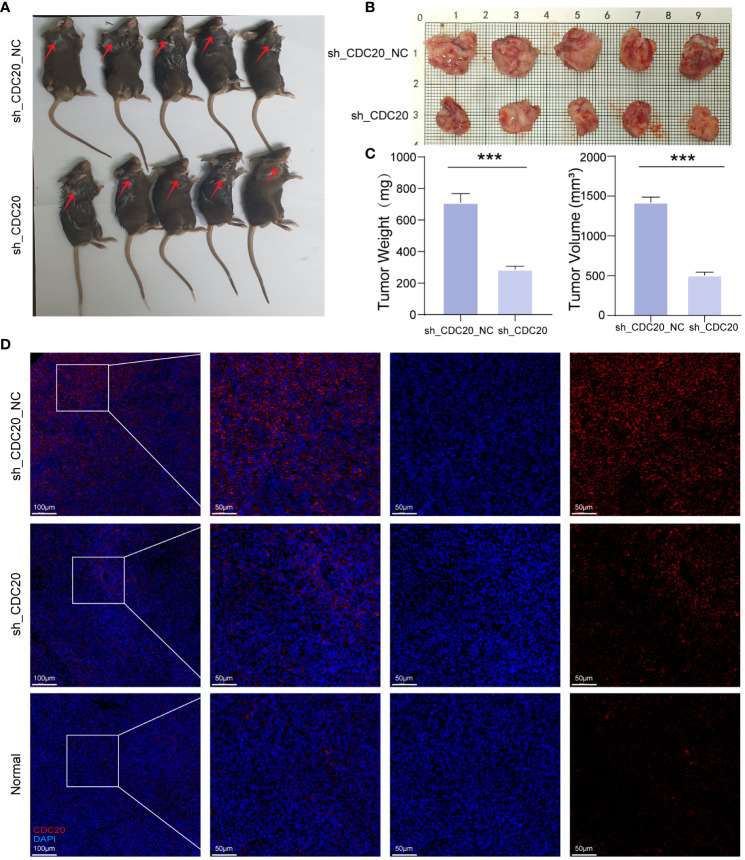
Knockdown of CDC20 inhibits tumor growth in mouse xenotransplantation models. **(A, B)** Images of mice and tumors in the NC group and shCDC20 group. **(C)** The mean tumor weight histogram and tumor volume histogram of the NC group and shCDC20 group were measured after injection. **(D)** Immunofluorescence of tumor CDC20 in NC group and shCDC20 group, and immunofluorescence of lung tissue in normal mice. (***p<0.001).

To further investigate the impact of CDC20 on tumor progression *in vivo*, a mouse xenograft model was established (**Figures 8A, B**). Tumor growth was monitored, and the shCDC20 group exhibited a significantly lower average tumor volume of 503.96 mm3 compared to the control group (p ≤ 0.001). As anticipated, the average tumor weight in the shCDC20 group was 0.424g less than that in the shNC group (p ≤ 0.001) (**Figure 8C**).

Immunofluorescence analysis of CDC20 expression in tumors from the shCDC20 and NC groups revealed a noticeable decrease in CDC20 fluorescence levels and expression in the shCDC20 group (**Figure 8D**). In addition, we conducted immunofluorescence detection of CDC20 in normal mouse lungs. Compared to normal lung tissue, the expression of CDC20 in mouse lung cancer tissues was significantly increased. After knocking down CDC20, the tumor volume and weight were significantly reduced, suggesting that CDC20 may affect the progression of lung cancer in mice. In conclusion, the results obtained *in vivo* validate the role of CDC20 downregulation in tumor initiation and progression in lung cancer.

## 4 Discussion

Starting from the invasion- and relapse-related gene set, this study conducted WGCNA analysis to focus on differential gene modules. Subsequently, two algorithms, random survival forest and random forest, were used to obtain the intersection of gene sets and select three key genes. Studies have found that an elevated mRNAsi (RNA-based stemness index) is found in metastatic tumors and is associated with tumor heterogeneity, which may explain its relationship with tumor progression, staging, treatment resistance, and poor prognosis (20). Therefore, this study further investigated the relationship between these key genes and mRNAsi to assess the degree of dedifferentiation in carcinogenesis, with a focus on the gene CDC20. Subsequently, the prognosis of CDC20 and its malignant clinical characteristics in lung adenocarcinoma (LUAD) patients were investigated, along with *in vivo* and *in vitro* experiments through CDC20 knockdown. The results of this study showed that the expression of CDC20 was positively correlated with the progression of LUAD. Loss of CDC20 suppressed the growth and migration of lung cancer cells by inhibiting the MAPK signaling pathway and induced cell cycle arrest. These results suggest that CDC20 can serve as a prognostic biomarker for LUAD and is a valuable new potential target for research.

Cell Division Cycle 20 (CDC20) is a key molecule that plays an important role in the cell cycle, including serving as a spindle assembly checkpoint and as an activator of the Anaphase-Promoting Complex/Cyclosome (APC/C) (21). It has been reported to be highly expressed in various malignant tumors and plays a critical role in tumor occurrence and progression (22–24). Studies have found that abnormal expression of CDC20 is associated with the malignant progression and poor prognosis of various types of cancer, including pancreatic ductal adenocarcinoma (25), gastric cancer (26), urothelial bladder carcinoma (27), astrocytoma (28), hepatocellular carcinoma (29), and oral squamous cell carcinoma (30). However, the potential molecular mechanism of CDC20 may be related to Scaffold Matrix Attachment Region-Binding Protein 1 (SMAR1), a tumor suppressor. Paul et al. suggested that CDC20 is responsible for maintaining high levels of SMAR1 in advanced cancer and that CDC20-mediated proteasomal degradation of SMAR1 promotes cell migration and invasion (31). According to reports, silencing of CDC20 inhibits the growth of prostate cancer and enhances sensitivity to docetaxel chemotherapy (32). Furthermore, overexpression of CDC20 enhances proliferation and invasion of pancreatic cancer cells (33). Research has also demonstrated that mouse embryos lacking CDC20 may experience a mid-term arrest (34). In support of its oncogenic function, CDC20 depletion leads to effective tumor regression (35). Conversely, the loss of spindle assembly checkpoint regulation by CDC20 promotes tumorigenic progression (36), suggesting that CDC20 may be a potential cancer treatment target.

In non-small cell lung cancer, although some studies have previously reported on CDC20 (37, 38), little is known about the relevance of these mechanisms in lung cancer development, and further molecular biology experiments and clinical validation are lacking. Therefore, we conducted a series of *in vitro* experiments on human lung cancer cell lines, including wound healing assays, Transwell migration assays, colony formation assays, and flow cytometric cell cycle analysis. The aim of these experiments was to investigate the function of CDC20, with a particular focus on whether CDC20 can affect the invasive and metastatic capabilities of cells.This study conducted *in vivo* and *in vitro* experiments by knocking down CDC20. The *in vitro* results showed that CDC20 knockdown effectively inhibited the invasion and migration of lung adenocarcinoma cells. Additionally, flow cytometry analysis revealed the regulatory role of CDC20 in the cell cycle of lung cancer cells, with CDC20 knockdown leading to S phase blockade.When the CDC20 gene is knocked out, the loss of CDC20 interferes with the proper assembly and function of the spindle, which in turn affects the normal progression of mitosis. Other studies have suggested that CDC20 is involved in the formation of E3 ubiquitin ligase by APC/C, controlling substrate specificity and degradation time. CDC20 primarily functions during the mid-late transition and mitotic exit (39, 40), while another key gene in the cell cycle, CDH1, has the opposite function, playing a role in the late mitotic phase and G1 phase (41, 42). In the metaphase to postmitosis transition period, CDC20 activates anaphase promoting complex (APC) promoting cell cycle in mitosis, and APC forms APCCDC20 complex through the activation of CDC20. This complex performs ubiquitination of securin and cyclin B, thus playing a crucial role in the mitosis process. If the CDC20 gene is knocked out, this ubiquitination process will not be able to proceed, resulting in the cells not being able to transition from the middle to the late stage, ultimately resulting in the arrest of the S phase, which was also confirmed by our flow cytometry results.

Simultaneously, we aim to validate the significant impact of CDC20 on lung cancer *in vivo*. Therefore, we selected the LA-4 mouse lung cancer cell line to investigate whether knocking down CDC20 would inhibit the growth of mouse lung tumors. *In vitro* experiments enable us to meticulously investigate the direct effects and regulatory mechanisms of CDC20 in human cancer cells, whereas *in vivo* experiments offer the opportunity to assess the impact and applicability of these mechanisms within a physiological environment. Utilizing mouse cancer cell lines *in vivo* models allows for a more accurate simulation of the biological processes of tumor growth and metastasis, while also facilitating genetic manipulation and monitoring.

Regarding the comparability of CDC20 levels between human and mouse cell lines, we acknowledge the potential for biological differences that preclude direct comparison between the two. However, as CDC20 is a protein that plays a crucial role in cell cycle regulation and is conserved in its function between humans and mice. In our studies, knocking down CDC20 consistently resulted in reduced expression in both human and mouse cell lines. Immunofluorescence results indicate that CDC20 is highly expressed in mouse lung cancer and its knockdown significantly reduces tumor size, indicating its involvement in tumor formation *in vivo*. We believe that the mouse tumorigenicity assay can assess the tumor-forming capacity of cells *in vivo*, a process that involves cell proliferation, survival, and local invasion. While the assessment of multi-organ metastasis is an important component of tumor research, local invasion is a prerequisite for metastasis. Studying local invasion can provide a foundation for understanding the mechanisms of metastasis. Therefore, the mouse tumorigenicity assay complements the cell migration/invasion assays; the former provides information on tumor growth and local invasion, while the latter helps to delve deeper into how cells acquire invasive and metastatic capabilities.

This study also discovered the significant involvement of the MAPK signaling pathway in cell migration and proliferation processes. The MAPK signaling pathway is capable of transducing extracellular signals into the cell, leading to the activation of downstream kinases and cascade reactions that regulate the expression of target genes. Relevant research has demonstrated that as the malignancy of tumors increases, the MAPK signaling pathway becomes activated, resulting in enhanced cell proliferation and migration abilities, ultimately exacerbating the severity of the disease (43). Therefore, targeting the reduction of CDC20 expression by inhibiting the MAPK signaling pathway may present a potential therapeutic approach for lung adenocarcinoma.

However, there are still some limitations in our study. Firstly, despite the utilization of multiple datasets and the removal of batch effects, there may still be lingering biases. Moreover, our research lacks in-depth exploration of CDC20, necessitating further investigation using technologies like single-cell sequencing and gene editing.Furthermore, *in vivo* experiments, we only observed the changes in CDC20 and did not further investigate the correlation and potential influences on other related genes or signaling pathways after knocking down CDC20. This aspect will be considered in our future studies to provide a more comprehensive and in-depth analysis.

## 5 Conclusion

This study identified the key gene CDC20 as a driver of lung adenocarcinoma recurrence and invasion through comprehensive bioinformatics analysis. Downregulating CDC20 expression allowed for targeted regulation of the MAPK signaling pathway, effectively inhibiting the invasion and migration abilities of lung adenocarcinoma cells. *In vivo* experiments demonstrated that knocking down CDC20 inhibited tumor formation. These findings suggest that CDC20 may serve as a crucial diagnostic and therapeutic target for post-operative recurrence or inhibition of tumor invasiveness in clinical lung adenocarcinoma patients.

## Data availability statement

The original contributions presented in the study are included in the article/supplementary material. Further inquiries can be directed to the corresponding authors.

## Ethics statement

The animal study was approved by the medical ethics committee of Anhui University of Science and Technology. The study was conducted in accordance with the local legislation and institutional requirements.

## Author contributions

RM: Data curation, Methodology, Writing – original draft. ZX: Data curation, Methodology, Writing – original draft. TH: Data curation, Methodology, Writing – original draft. YL: Writing – review & editing. JZ: Writing – review & editing. JG: Writing – review & editing. YX: Writing – review & editing. YB: Writing – review & editing. ZH: Writing – review & editing. JW: Conceptualization, Supervision, Writing – review & editing. WW: Conceptualization, Supervision, Writing – review & editing. DH: Conceptualization, Supervision, Writing – review & editing.
